# The history of the elimination of lymphatic filariasis in China

**DOI:** 10.1186/2049-9957-2-30

**Published:** 2013-12-02

**Authors:** Sun De-jian, Deng Xu-li, Duan Ji-hui

**Affiliations:** 1 Institute of Parasitic Diseases, Chinese Center for Disease Control and Prevention, PO Box 200025, Shanghai, China; 2 Shandong Institute of Parasitic Diseases, PO Box 272133 Jining, China; 3 Hunan Provincial Center for Disease Control and Prevention, PO Box 410005, Changsha, China

**Keywords:** Lymphatic filariasis, Control strategies, Elimination

## Abstract

China used to be one of the most heavily endemic countries for lymphatic filariasis (LF) in the world. There were 864 endemic counties/cities in 16 provinces/autonomous regions/municipalities (P/A/M) with a total population of 330 million at risk of infection. Since the founding of the People’s Republic of China in 1949, the Chinese Government has designated the control of the disease to be a top priority. Due to decades of sustained efforts, close cooperation related to LF control among government departments, and active participation of endemic populations, an all-round campaign for prevention and control has been carried out vigorously and successfully. Over many years, great achievements have been made through persistent endeavors of Chinese scientists and disease control workers. The ultimate goal to eliminate LF in the country was achieved in 2006.

## Multilingual abstract

Please see Additional file 1 for translation of the abstract into the six official working languages of the United Nations.

## Review

Lymphatic filariasis (LF) puts more than a billion people in more than 80 countries at risk of infection. The symptoms and signs of LF, including acute inflammatory episodes, as well as the progressive chronic diseases of lymphedema, chyluria and hydrocele, caused tremendous suffering and overall disability. The patients and their family members suffered from physiological and psychological pain. The prevalence of LF seriously affected economic development and social stability. The global program for the elimination of LF was launched in 2000 following a resolution of the Fiftieth World Health Assembly, which was to eliminate LF as a public health problem worldwide.

China used to be one of the most heavily epidemic counties for LF in the word. There were 864 endemic counties/cities in 16 provinces/autonomous regions/municipalities (P/A/M) with a total population of 330 million at risk of infection in the 1980s. Since the founding of the P. R. China in 1949, the Chinese Government has designated the control of the disease to be a top priority. Due to decades of sustained efforts, close cooperation related to LF control among government departments, and active participation of endemic populations, an all-round campaign for prevention and control has been carried out vigorously and successfully. Over many years, great achievements have been made through persistent endeavors of Chinese scientists and disease control workers. The ultimate goal to eliminate LF in the country was achieved in 2006.

By demonstrating the serious harm of LF, the great importance attached by the Government, specific control goals, sound control and monitoring networks, and scientific research assisted prevention and treatment, this paper serves as a documentary of the arduous process and experiences of China’s LF control program.

## History of lymphatic filariasis in China

### Ancient times

Lymphatic filariasis (LF) is an ancient parasitic disease. Although no such term as “filariasis” was mentioned in ancient Chinese medical literature, symptoms similar to the manifestations of LF were recorded in some early Chinese traditional medical books, the earliest being in 600–700 B.C. At the time of the Sui Dynasty, a detailed description of symptoms similar to the manifestations of LF including filarial acute lymphadenitis/lymphangitis (ADL), lymphedema/elephantiasis, chyluria, and hydrocele was recorded in *General treatise on the cause and symptoms of diseases* published in 610 A.D by the famous ancient physician Chao Yuanfang [1]. More detailed records of the four manifestations mentioned above were kept by some ancient physicians during the Tang, Song, Jin, Yuan, Ming, and Qing dynasties. The areas mentioned were mainly along the eastern Yangtze River and in some parts of southern China. This is quite in accordance with the more recent distribution of LF in the country [2].

### Scientific discoveries

In recent scientific literature, Dr. Patrick Manson reported that there was elephantiasis of the scrotum in Xiamen, south of the Fujian province, in 1872 [3]. He described microfilariae of *Wuchereria bancrofti*, microfilarial of sheath, and a female adult of *W. bancrofti* in 1876 [4]. In 1877, he found that the number of microfilariae examined at night was more than that in the daytime. In 1881, Dr. Manson confirmed again that microfilaria appeared in the circulating blood only at night by examining two cases every three hours for 23 days. Rennie also confirmed the nocturnal periodicity in Fuzhou, middle of the Fujian province, in the same year [5]. Between 1878 and 1882, Dr. Manson found that the *Culex quinquefasciatus* was the intermediate host and vector of microfilariae, when he was studying the relationship between microfilariae and elephantiasis [4,6].

Besides the work of Dr. Manson, some information about the prevalence of bancroftian filariasis in China was reported by foreign physicians. Meadows [7] reported that there was a lot of elephantiasis in the Zhejiang province. Whyte [8] found that the disease, which sometimes caused eosinophilia, was distributed in Chaozhou, Guangdong province. Maxwell [9] reported that filariasis was frequently found in the east of the Fujian province during the 20-year period that he was a doctor in Fujian.

The first survey of filariasis by Chinese investigators was carried out by Li Zhongen in 1925, which showed that bancroftian filariasis was widely prevalent in the north of the Jiangsu province [10]. A case of brugian filariasis that came from Wenzhou, Zhejiang province was first reported by Feng Lanzhou in 1933 in Xiamen, south of the Fujian province [11]. Feng Lanzhou and Yao Kefang also found *Brugia malayi* in Huzhou, Zhejiang province in 1935 [12]. Brugian filariasis in the Fujian province was also reported by Hu Meiji in 1937 [13] and Chen Guozhong in 1948 [14]. These surveys were carried out among the patients and hospital staff in cities and counties, and field surveys on filariasis were very limited before 1949, the year of the establishment of new China. However, through experimental and field studies, some progress has been made by the Chinese investigators. This includes a preliminary understanding of the epidemiology of filariasis, confirmation of the existence of both *W. bancrofti* and *B. malayi,* and identification of the principal vectors for *B. malayi, Anopheles sinensis, W. bancrofti, Culex quinquefasciatus*, and *C. pallens.*

## Epidemiological surveys of LF

### Distribution

**Figure 1:**
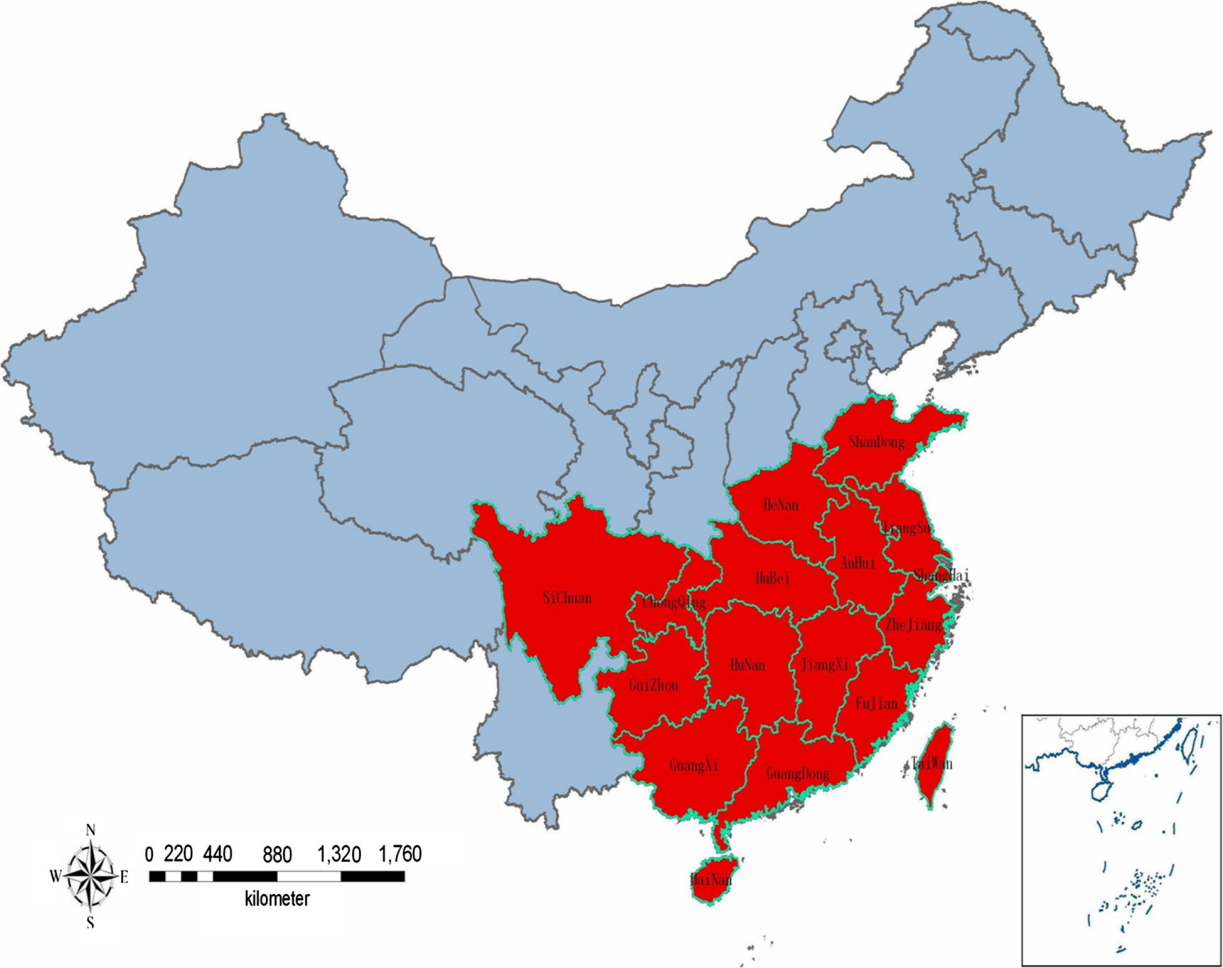
Distribution of endemic areas for LF in China (in red).

By the mid 1950s, the basic distribution and epidemiological features of both bancroftian and brugian filariasis had been established [15]. Up to the early 1980s, through two large-scale surveys started in the late 1950s and early 1970s, it was confirmed that the disease was widespread in 16 provinces/autonomous regions/municipalities (P/A/M), i.e., Shandong, Henan, Hubei, Anhui, Jiangsu, Shanghai, Zhejiang, Jiangxi, Fujian, Guangdong, Hainan, Hunan, Guangxi, Guizhou, Sichuan, and Chongqing, which included 864 counties/cities. Of these, 463 counties/cities had bancroftian filariasis, 217 had brugian filariasis, and 184 had mixed infection of the two. Of the 16 endemic P/A/M, Shandong, Hainan, and Chongqing had bancroftian filariasis only; Sichuan had brugian filariasis only; and the other 12 had mixed infection (see Figure 1 and Table 1) [2].

**Table 1:** The distribution of LF and filarial species in China*

**Province/autonomous region/municipality**	**No. of endemic counties/cities**	**Species**		
		***W. bancrofti***	***B. malayi***	**Mixed infection**
Shandong	74	74		
Henan	71	42	1	28
Hubei	69	10	47	12
Anhui	82	28	14	40
Jiangsu	71	64	0	7
Shanghai	10	5	2	3
Zhejiang	67	3	40	24
Jiangxi	75	9	33	33
Fujian	68	16	24	28
Guangdong	62	61	1	
Hainan	18	18		
Hunan	55	39	10	6
Guangxi	68	60	6	2
Guizhou	47	21	25	1
Sichuan	14		14	
Chongqing	13	13		
Total	864	463	217	184

*Taiwan is not included in this table.

The distribution of bancroftian filariasis extended from Lelin county of the Shandong province in the north (37°48′N) to Sanya city of the Hainan province in the south (18°10′N), and from Zhoushan archipelago of the Zhejiang province in the east (122°30′E) to Tongzi county of the Guizhou province in the west (106°50′E). The distribution of brugian filariasis extended from Bo’ai county of the Henan province in the north (35°21′N) to Loucheng county of the Guangxi Zhuang autonomous region in the south (24°50′N), and from Zhoushan archipelago of the Zhejiang province in the east (122°30′E) to Ya’an city of the Sichuan province in the west (103°E) [2].

Prof. Fan Ping-Chin [16] reviewed that bancroftian filariasis was also prevalent in Yunlin, Jiayi, Tainan, Gaoxiong and Pingtung counties in Taiwan, and Jinmen, Penghu and Matsu Islands, and that the disease in these areas has been controlled effectively.

### Vector mosquitoes

Through nation-wide investigations on mosquito fauna in human habitat, bionomics and behavior of common mosquitoes, and natural and artificial infections with *W. bancrofti* and *B. malayi*, it was found that there were 16 species/subspecies in four genera of potential vectors transmitting two species of filarial parasite. Of these, two species of culicine mosquitoes, *C. pallens* and *C. quinquefasciatus,* were most important in the epidemiology of bancroftian filariasis, while two species of anopheline mosquitoes, *A. sinensis* and *A. anthropophagus*, were most important for brugian filariasis [2].

#### Vectors of bancroftian filariasis

*Culex pallens* (*Culex pipiens pallens* Coquillent, 1898) and *Culex quinquefasciatus* (*Culex pipiens quinquefasciatus* Say, 1821) are two geographical subspecies of *Culex pipiens* (Linnaeus, 1758). The former is mainly distributed to the north of 32°N, while the latter is found to the south of 32°N. The morphology of the two species is similar. Both *C. pallens* and *C. quinquefasciatus* adults prefer human blood. The main resting place of *C. pallens* and *C. quinquefasciatus* is in human dwellings. Their larvae mainly breed in the small water collections around human dwellings and in containers, especially with foul water.

The results of investigations of natural infection of *C. pallens* showed that the highest positive rate with filarial larvae was 44.9% (1957) and the highest positive rate with infective larvae (L3) was 16.1% [17]. Meanwhile in *C. quinquefasciatus*, the highest positive rate with filarial larvae was 38.8% (1964) and the highest positive rate with L3 (1958) was 7.5% [18].

#### Vectors of brugian filariasis

*A. sinensis* and *A. anthropophagus* are two of the members of the *A. hyrcanus* group. The egg “deck” of *A. sinensis* is broad, taking up about 45% of the width of the egg (including the floating chamber). The larvae of *A. sinensis* breed in broad and static water bodies that are exposed to sunlight, and are slightly polluted and warm. The main breeding places are paddy fields, seedling plots, reed marshes, lotus pools, irrigation ditches, etc. The adult *A. sinensis* prefers animal blood, but also feeds on humans. *A. sinensis* is a semi-indoor resting mosquito species, which usually intrudes into human dwellings or cattle sheds at night for a blood meal and flies away at dawn, with only a few staying behind [2]. *A. sinensis* is highly susceptible to *B. malayi* and is distributed in nearly all the provinces in China, except Qinghai and Xinjiang. Based on investigations in different areas, the positive rate of natural infection with *B. malayi,* L3 was 0.3–6.8% (1968) and the positive rate of experimental infection with L3 was 71.7–95.7% [19-22].

*A. anthropophagus* is also named *A. lestri var anthropophagus*[23]. The morphology of adults and larvae is similar to that of *A. sinensis.* The egg “deck” is narrow, taking up about 10% of the egg width. *A. anthropophagus* prefers to breed in static water shaded by aquatic vegetation, where the water is clean, cool, and sandy. The main breeding places are paddy fields, irrigation ditches, small pools, wild rice stem fields, reed marshes, springs, etc. Most of the larvae breeding in paddy fields scatter in the shaded places around the root of rice, usually together with the larvae of *A. sinensis. A. anthropophagus* prefers human blood and is and indoor-resting species. Most of the females stay indoors after a blood meal. The range of distribution of *A. anthropophagus* is not as extensive as that of *A. sinensis. A. anthropophagus* is highly susceptible to *B. malayi.* The results of surveys in different areas of China indicated that the positive rate of natural infection with L3 was 3.0–32.4% (1957–1959) [24]^,^ significantly higher than that of *A. sinensis.* Its positive rate of artificial infection with L3 was 71.4–95.5% [21,22], similar to that of *A. sinensis.* However, due to its preference for human blood, it is more dangerous than *A. sinensis.*

In artificial infection, *A. anthropophagus* is significantly more susceptible to bancroftian filariasis than *A. sinensis*[25]. The result of investigations on its natural infection in Shucheng county of the Anhui province demonstrated that *A. anthropophagus* was one of the vectors transmitting bancroftian filariasis locally [26].

### Prevalence

Among the 864 counties/cities endemic for LF in China, 542 were hypo-endemic areas (microfilaria [MF] rate ≤ 5%) and 287 were meso-endemic areas (MF rate 5%- ≤ 20%), while 33 were hyper-endemic (MF rate 20%- ≤ 30%) and two were super-endemic areas (MF rate>30%) [2].

The total population at risk of LF in endemic areas was about 330 million. It was estimated that the total number of filariasis cases was 31 million (including microfilaremics and clinical cases), of which 22 million were bancroftian and nine million were brugian filariasis, i.e., about two-thirds of cases in China were bancroftian in the 1980s [2].

The data from investigations in different areas showed that the degree of prevalence of LF was related to economic conditions, hygienic status, human behavior, geographical environment, species of mosquito vector, and filaria. In areas where economic and sanitary conditions were poor and people slept outdoors in the summer, the MF rate was very high. For example, before the founding of the People’s Republic of China, Fengyang County in the Anhui province suffered from frequent floods, and its MF rate of *W. bancrofti* was as high as 35.4%, the highest among the LF endemic areas in China.

### Clinical manifestations

Lymphadenitis/lymphangitis (ADL) are common acute clinical manifestations of bancroftian and brugian filariasis. *W. bancrofti* parasitizes in the superficial and deep lymphatic systems, while *B. malayi* parasitizes in the shallow lymphatic system only.

*W. bancrofti* usually parasitizes in the genitourinary lymphatic system, while *B. malayi* mainly in the lymphatics of limbs. The difference in parasitic sites between the two species of filariae could produce ADL at distinct parts of the body. Varicophlebitis, epididymitis, and orchitis are seen in acute bancroftian filariasis, and often occur at the same time.

Hydrocele and chyluria are the common chronic symptoms of bancroftian filariasis. Lymphedema/elephantiasis is common signs observed both in bancroftian and brugian filariasis. In bancroftian filariasis, leg elephantiasis usually extends over the whole leg, forming huge elephantiasis. In brugian filariasis, elephantiasis is limited to the limbs, and leg elephantiasis seldom expands beyond the knee level. The elephantiasis in the upper limbs is rare and mostly restricted to below the elbow joint [2].

## Control activities

### Strategies for control of LF

In general, three strategies for the control of vector-borne diseases can be considered: vector control, elimination of infection sources, and the integration of vector control with elimination of infection sources. In the 1950–1960s, the majority of experts considered integrated measures as the best strategy for LF control. However, since the disease was widely endemic in P.R. China, and the integrated measures needed lots of time, manpower and material resources, it seemed very difficult to put the measures into practice. Then, Prof. Feng Lanzhou (1962), the former director of the Institute of Parasitic Diseases (IPD) at the Chinese Academy of Medical Sciences (CAMS), pointed out that the pathogen biology of filariasis was very different from that of other vector-borne diseases [27]. In fact, the parasite has many vulnerable points which can be exploited for the control of the disease. These are: ① After entering the mosquito, a microfilaria (MF) can grow but not multiply and the probability of it growing into an infective larva is less than 1, and ② the probability of the infective larva within a mosquito getting into a human and growing into its adult stage is also less than 1. Microfilaria can only be reproduced when both male and female worms are present, and after they have copulated. Therefore, only when a number of infective larvae enter the same individual, then this person will have a patent infection (with MF in his/her blood), and can be an infection source. The traditional antifilarial drug, diethylcarbamazine (DEC), can affect both adult worms and MF, and reduce infection sources. In China [28], there are no animal reservoir hosts for both of *W. bancrofti* and *B. malayi.*

In the early stage of LF control, some institutions of research and control, such as the Shandong Provincial Institute of Parasitic Diseases, the Guangxi Institute of Parasitic Diseases, and IPD at CAMS, conducted field trials on anti-filariasis strategies and intervention measures for several consecutive years. The results demonstrated that repeated blood surveys and DEC treatment of microfilaremia and/or mass chemotherapy of the whole population in the village could eliminate the infective sources, as well as integrated measures, and could reduce the MF rate to below 1% [29-31].

Both the experimental research in the field and the large-scale control practice proved that the strategy of eliminating infection sources could control LF effectively. Thus, in the early 1970s, the strategy of eliminating infection sources as the principle measure in LF control in China was established. This procedure greatly promoted the process of LF control in the country.

### Control schemes with DEC

There were three schemes with DEC for LF control in China: ① Repeated blood surveys and treatments, ② treatment of microfilaremia cases combined with mass chemotherapy of the whole population in an endemic area, and ③ treatment of microfilaremia cases, integrated with DEC salt.

#### Repeated blood surveys and treatments

Repeated blood surveys in the whole population plus treatments of microfilaremia cases were called “target treatment”, and were widely adopted in the early stage of LF control in China. Based on experience, in 1981, the LF control program recommended repeated blood surveys and treatments in brugian filariasis endemic areas and hypo-endemic areas of bancroftian filariasis. The DEC treatment regimen for bancroftian filariasis was 3.0 g in three to five days, or 4.2 g in seven days, given two to four times at one-month intervals. For brugian filariasis, the recommended dosage was 1.5–2.0 g in two to three days, given two to four times at one-month intervals. Generally, blood surveys and treatments should be conducted two to three times [32].

#### Target treatment combined with mass chemotherapy of the whole population in endemic villages

Through repeated blood surveys and treatments, it is possible to reduce the MF rate of the population to a low level, even to less than 1%, but it needs to be carried out over a rather long time period. The reasons are many-sided and the most important one is the low sensitivity of the blood smear method presently used. Low-density microfilaremia can be easily missed and prepatent cases cannot be detected since there is no MF in their blood. In order to improve the effectiveness of the LF treatment and to speed up control, studies on the effects of mass chemotherapy were carried out by several institutions in the early 1960s. The regimen was recommended in meso- and hyper-endemic areas of bancroftian filariasis, and also in hyper-endemic areas of brugian filariasis. The practical dosage for the MF-negative residents above five years old was one course of 3 g DEC in five days or 4.2 g in seven days in bancroftian endemic areas, and one course of 1.5–2.0 g DEC in two to three days in brugian endemic areas. For the treatment of microfilaremia cases, the dose and the course were the same as in the repeated blood surveys and treatments, recommended in 1981 by the filariasis control program of China [32].

#### Target treatment for MF carriers integrated with DEC salt

The biggest advantage of using DEC fortified salt (DEC salt) was that rare and, if any, very mild, side effects occurred, for which most of the patients did not need special treatments. In 1972, the Shandong Institute of Parasitic Diseases first tried this regimen in hyper-endemic areas of bancroftian filariasis. In two villages of the Tengxian County, Shandong province, the effects of DEC salt were observed for ten years. Before the treatment, the MF rate was 8.89%, which dropped to 0.63% immediately after the six-month DEC salt treatment, and no new infection was found in the following ten years [33]. Following Shandong province, pilot trials on DEC salt were launched one after another in the Jiangsu, Henan, Guangxi, Guizhou, Zhejiang, Anhui, Hunan, and Guangdong provinces; and from the early 1980s, the regimen was quickly adopted by 15 P/A/M [34].

Filariasis control by means of DEC salt in a large area of China experienced the following: ① When the simple DEC salt (meaning DEC salt alone) was applied in original meso- or hyper-endemic areas, where the MF rate had reduced significantly by the repeated blood surveys and treatments, or in hypo-endemic areas where blood survey and treatment had not been undertaken, the total dosage of DEC and the length of time for the treatment could be determined according to MF rate and density. Generally, the concentration of DEC salt was 0.3%, and the duration of treatment could be three, four and a half, or six months to obtain the total dosage of 4.5 g, 6 g, and 9 g/person, respectively. The regimen was not only effective in LF control, but in economic terms of manpower, materials, and money; ② When the target treatment combined with DEC salt was chosen as a control measure in the meso- and hyper-endemic areas of bancroftian filariasis, where survey and treatment had not been conducted, the recommended schedule was to perform a general blood survey first, then to treat the whole population with DEC salt, and finally to carry out the target treatment. These procedures could reduce side effects. In general, the concentration of DEC salt was 0.3%. The duration of time for taking DEC salt depended upon the MF rate and the level of microfilaremia. Generally, it took four to six months with a total dosage of 6 to 9 g DEC/person. The regimen for the target treatment was the same as that in the repeated blood survey and treatment; ③ When the simple DEC salt was applied in the meso- or hyper-endemic areas of bancroftian filariasis where no general blood survey and treatment had been performed, it was recommended that spot checks for microfilaremia should be done in selected places at first, in order to understand the MF rate and density in the locality. Based on the data, the total dosage and the duration of treatment could be determined. Generally speaking, the total dosage of 0.3% DEC was 9 g/person, but may be increased up to 13.5 g or even more. Two to four courses (three-month treatment) were given intermittently to get good effects. In a hyper-endemic area, where a portion of MF-positive people had comparatively high MF counts, the total dosage of 9 g DEC could not be sufficient to turn the high-density microfilaremia cases negative [32,34,35].

The use of DEC salt was not very suitable in the coastal region of China because there were many salt works and it was very difficult to control the salt sold by private merchants. Residents in some districts liked to use soy sauce or shrimp soy instead of cooking salt. Under such circumstances, DEC was mixed with other salt products e.g., DEC soy or DEC shrimp soy to get the same control effect [36].

### Pros and cons of the three control schemes

Comparing the three control schemes discussed above, the target treatment can save drug, but even repeated night blood surveys are unable to find out all the microfilaremia cases, and thus multiple treatments cannot cure all MF positives. The scheme needs a longer time period, and more manpower and money. For the control program long-term, repeated surveys and treatments will make people, as well as disease control workers, tired of the activities, and result in little enthusiasm and morale [37]. The target treatment combined with mass chemotherapy can result in good effects quickly. However, the success depends upon a high drug intake rate. The large workload for drug delivery, especially when people live scattered in mountainous regions, is a big shortcoming. Also, it is difficult to confirm that the full dosages were actually taken by people. In addition, attention has to be paid to possible acute abdomen among children due to ascarids after DEC treatment. The merits of DEC salt are its rare side reactions and safety. These advantages solve the problem of low acceptability by people experienced with the other two regimens. The scheme can also reduce difficulty in drug delivery, thus saving lots of manpower [24]. The scheme with DEC salt, however, requires a huge amount of DEC. The regimen of target treatment combined with mass chemotherapy or DEC salt is suitable for the meso- and hyper-endemic areas to make up for the weakness of the target treatment, and to reduce the MF rate of the population quickly.

According to the data accumulated in the entire control stage from 1956 to 1994 in China, 720.2 million person-times accepted the blood survey and 23.3 million were MF positive. Filariasis control with DEC was carried out for 213.5 million person-times, among which 22.1 million accepted target treatment, 33.6 million accepted mass chemotherapy, and 157.8 million took DEC salt. The number of people who took DEC salt accounted for 73.9% of the total treated person-times, and it is obvious that DEC salt played a very important role in the control of filariasis in P.R. China [34].

## ‘Basic elimination’ and elimination of LF

### ‘Basic elimination’ of LF

In 1983, the Ministry of Health (MOH) issued a document entitled *Criteria for the basic elimination of filariasis and assessment methods*. The main criterion was ‘in a filariasis prevalent county/city, through intervention, the MF rate was reduced to below 1% in the population of administrative villages.’ In China, the assessment of basic elimination of filariasis was carried out at three levels: self-assessment by the county, spot checks of the results of counties by the provinces, and spot checks of the provincial results by the MOH. The composition of the evaluation group, the evaluated range, the number of persons to be checked, sites to be selected, and quality control were all assigned [38]. After the 1980s, the number of counties/cities assessed at the provincial level, and which reached the criteria of basic elimination of filariasis, increased yearly. At the end of 1985, 660 counties/cities had basically eliminated filariasis, which made up 76.4% of the total 864 endemic counties/cities [39]. By 1994, all 864 endemic counties/cities of the 16 P/A/M had reached the basic elimination criteria.

From 1981 to 1994, the MOH organized evaluation in Shanghai (1981), Shandong (1983), Guizhou (1984), Guangxi (1985), Sichuan (including Chongqing) and Hunan (1986), Henan, Guangdong and Hainan (1987), Hubei and Fujian (1988), Zhejiang, Jiangsu and Jiangxi (1989), and Anhui (1994), altogether 16 P/A/M, to assess the basic elimination of filariasis. Blood examinations were performed on 204,508 persons in 192 villages of 36 counties in 12 P/A/M, and 54 persons were positive (average MF rate: 0.03%, range: 0–0.51%) [34,39]. The MOH declared that these P/A/M had reached the criteria of basic elimination of filariasis.

### Transmission threshold at which LF disappears spontaneously

In 1982, the MOH approved the study on the transmission threshold of filariasis as a key project, and a nation-wide collaboration was developed with a unified design and method. Twenty-one villages from 11 provinces with a total population of 32,396 were selected for the study. The microfilaria rate in the population was approximately 0.5% in each of the seven villages (six bancroftian and one brugian infections), 1.0% in eight villages (three bancroftian, four brugian, and one mixed infection), 1.5% in five villages (four bancroftian and one brugian infections), and 2.5% in one village (*B. malayi* infection). In the 21 research villages, there were 349 microfilaremia cases. The microfilaria density was below 5/60 μl in nine villages (five bancroftian and four brugian infections), 5–10/60 μl in eight vi1lages (six bancroftian and two brugian infections), and over 10/60 μl in four villages (two bancroftian, one brugian, and one mixed infections). No control measures were taken in these villages, and follow-up blood surveys for residual microfilaremia were done every year or every two years. Mosquitoes were collected and dissected, and changes in larval infection rates were observed in relation to human microfilaria rates. In the transmission season, surveys on mosquito biting density and the use of mosquito nets were carried out [40].

In 1994, data from the collaborative studies in the 21 villages were put together. The results demonstrated that, under the circumstances where the man-biting density of mosquito vectors was within 20–50 mosquitoes/person/night, and about 40% of the population used bed-nets seasonally, the MF rate was ≤1.71% in areas with bancroftian filariasis and ≤1.5% in areas with brugian filariasis. The mean microfilaria density of residual microfilaremia cases in most villages was 3–10/60 μl blood. It was considered that filariasis transmission in these villages had already dropped below the critical threshold level and that the transmission was virtually interrupted [41].

### Surveillance for the assessment of LF elimination

In 1988, the MOH issued a document entitled *Technical scheme for surveillance work in areas of basic elimination of filariasis*. The document’s aim was to standardize the surveillance work in the whole country and to promote the development of the work. Both theoretical knowledge and control practice indicated that filariasis had two characters prone to its interruption and ultimate elimination. First, filariasis transmission efficiency was low and the residual low-density microfilaremia cases had no epidemiological significance. Second, if the basic elimination of filariasis had once been achieved, the residual infection sources would, without treatment, gradually disappear in a few years (four to five years for brugian filariasis, and five to seven years for bancroftian filariasis). It was considered unnecessary to carry out a general blood survey to find out the residual infection sources and treat them. Elimination of filariasis was possible through systemic surveillance over a period of time [32].

In China, “surveillance of filariasis” was unique and different from that of other diseases in that it was performed in places of basic elimination, where the MF rate and density of the population reduced to a very low level. The surveillance system included three parts: longitudinal surveillance, cross-sectional surveillance, and floating population surveillance. ① Longitudinal surveillance: Data were collected every two years in a fixed location with a population of about 1,000–2,000, where the MF rate and density used to be relatively high before intervention. The MF positive cases would not be treated. The aim of the longitudinal surveillance, which included mosquito studies, was to observe the dynamics of transmission of filariasis. ② Cross-sectional surveillance: An administrative village (with 1,000–2,000 people) was a unit for surveillance. Based on the original prevalence of filariasis and the locality of sample villages, a stratified cluster sampling method was used to select the subjects for the blood survey. The number of people to be examined should be more than 3% of the population of the county. The main aim of cross-sectional surveillance was, from multiple cross-sections, to observe the change in MF rate and density in the locality to provide bases for the elimination of filariasis. The microfilaremia cases observed should be treated by DEC. ③ Floating population surveillance: This was done in areas where the population migrated frequently. For those coming from an endemic area or living in the local place for at least six months, the blood examination or serological test for filariasis should be done to accumulate data and evaluate the impact of floating populations on the filariasis elimination program at different stages. Observed microfilaremia cases should be treated regularly [32].

According to the data accumulated between 1980 and 2004, from 82 longitudinal surveillance spots of 14 P/A/M, 69,018 persons were examined for MF in the first year and 196 residual microfilaremia cases were found. After the termination of control measures, the majority of residual microfilaremia cases turned to be negative one after another in the period of ten years. In the fifth and ninth year, no microfilaremia case was found for bancroftian and brugian filariasis spots, respectively, in the surveillance. In the first year of surveillance, 137,089 mosquitoes were dissected and 179 were positive for filarial larvae. The positive rate of mosquitoes decreased gradually, and after the eight year of the surveillance, no positive mosquito was found [42].

Summing up the parasitological data in cross-sectional surveillance conducted in 16 endemic P/A/M between 1972 and 2005, altogether 33,154,172 persons received blood examination, and 21,710 residual microfilaremia cases were found. When these data were analyzed according to the number of years after basic elimination of filariasis, 8,012 residual cases, 36.9% of the total MF positives, were observed in the first year (MF rate: 0.17%). In the 14^th^ year, there were five positives (MF rate: 0.0008%), and between the 15^th^ and the 28^th^ year, no residual cases could be found. From 1976 to 2005, cross-sectional mosquito vector surveys were performed. A total of 4.7 million mosquitoes were dissected and 809 filarial larvae positive mosquitoes were found. No positive mosquito was found since 1993 [42].

From 1984 to 2005, surveillance of the floating population was carried out. The total number of 363,485 persons was surveyed by blood examination and 419 microfilaremia cases were observed, all of them being detected before 1992. The results indicated that the probability of detecting microfilaremia cases in the floating population reduced greatly since all the original filariasis endemic counties/cities reached the level of basic elimination [42].

### Certification of LF elimination

In 1994, the MOH formulated two documents: *Criteria for the filariasis elimination (preliminary)* and *Evaluation of filariasis elimination (preliminary),* and issued them formally for practical use in 1996. The criteria for filariasis elimination were: regarding a county or an equivalent administrative region as a unit, through provincial level assessment, filariasis transmission has been interrupted (basic elimination of the disease) for more than ten years as a prerequisite; ① parasitological surveillance covering more than 3% of the total population in more than 30% of the total endemic towns/townships in a unit does not detect a microfilaremia case, and ② by mosquito-vector surveys, no human filarial larva is found in mosquitoes [38].

The evaluation of LF elimination was performed at prefecture and provincial levels. The overall data related to the surveillance and the original records of the control program were reviewed and evaluated. Spot checks of blood examination were performed when needed. From 1995 to 2006, the MOH organized evaluation in Guangxi (1995), Guizhou (1996), Shanghai (1996), Sichuan (1997), Chongqing (1997), Hunan (1997), Jiangsu (1998), Guangdong (2000), Hubei (2001), Zhejiang (2001), Fujian (2002), Shandong (2004), Henan (2004), Jiangxi (2005), Hainan (2005) and Anhui (2006), altogether 16 P/A/M, to assess the elimination of filariasis [43].

In March 2006, in the Fourth Meeting of the Global Alliance to Eliminate Lymphatic Filariasis held in Fiji, the Chinese MOH officially submitted the *National report on elimination of lymphatic filariasis in China* to the World Health Organization (WHO). In May 2007, the minister of MOH received a letter from Dr. Margaret Chan, the Director-General of WHO, confirming that China had reached the criteria for elimination of lymphatic filariasis as a public health problem.

## Conclusion

After half a century of diligent and sustained efforts that depended upon the proper strategies and effective measures to control the disease, lymphatic filariasis (LF), once heavily endemic in large areas of China and causing severe illnesses in the population had finally been wiped out. This is the result of the special attention paid by the Chinese Government towards the disease, as well as the continuous, active control. This success is also attributed to the long, hard struggle to control LF endured by research professionals over many generations. Elimination of LF is not only a great accomplishment in the field of public health in China, but also a brilliant model for the elimination of LF in other parts of the world.

Lymphatic filariasis is now widely endemic in the world, and a number of chronic cases still exist in China as a result of past infection. Strengthening the surveillance of imported sources of infection, as well as providing medical care for chronic cases, are long-term and difficult tasks for the control of LF.

## Competing interests

The authors declare that they have no competing interests.

## Authors’ contributions

SDJ contributed to the conception and drafting of the manuscript, and DXL and DJH contributed to the conception and writing of the manuscript. All authors read and approved the final manuscript.

## Supplementary Material

Additional file 1 Multilingual abstracts in the six official working languages of the United Nations. Click here for file

## References

[B1] Chao YF General Treatise on the Cause and Symptoms of Diseases 1956 Beijing: The People’s Medical Publishing House

[B2] Wang Z Control of Lymphatic Filariasis in China 1997 Beijing: The People’s Medical Publishing House 5 70

[B3] Dr Manson P Elephantiasis scroti Chin Customs Med Rep 1872 1 24

[B4] Liu XJ The discovery of filariasis in Fujian Chin J Med History 1982 12 3 155 158

[B5] Rennie T Report on the Health of Foochow for the year ended 31th March, 1881 Chin Customs Med Rep 1881 2 21 50 56

[B6] Dr Manson P On the development of *Filaria sanguinis hominis*, and on the mosquito, considered as a nurse J Linn Soc Zool 1878 14 75 304 311 10.1111/j.1096-3642.1878.tb01837.x

[B7] Meadow R Report on the health of Ningpo Chin Customs Med Rep 1871 1 35

[B8] Whyte GD Filarial periodicity and its association with eosinophilia J Trop Med Hyg 1909 12 176

[B9] Maxwell JP Filariasis in China Philip J Sci 1921 19 257 327

[B10] Lee CU Filariasis investigations in the Province of Kiangsu, China Tran Roy Soc Trop Med Hyg 1926 20 279 10.1016/S0035-9203(26)92302-X

[B11] Feng LZ A Comparative study of the anatomy of Microfilaria malayi Brug, 1927 and Microfilaria bancrofti Cobbold, 1877 Chin Med J 1933 47 1215

[B12] Feng LZ The distribution and transmission of filariasis in China Acta Cono Ter Trop Malar Morb 1938 1 239 248

[B13] Hu SMK A brief survey of filariasis in Foochow and Futsing regions, South China Chin Med J 1937 52 571 578

[B14] Chen KC A note on the filarial survey in Fukien province Lingnan Sci J 1948 22 85 92

[B15] Qian XZ Make great efforts for eliminating the five major parasitic diseases. Selection of the National Conference of Five Major Parasitic Diseases Control: Comprehensive data 1959 Beijing: The People’s Medical Publishing House 9

[B16] Fan PC A review of studies on filariasis in Taiwan with emphasis on filariasis eradication on Kinmen islands. Commemorative proceedings of honorary professor Fan Pingchin in his 81’s birthday 2003 Taipei: Yangming University 71 120

[B17] Sun HB Wang YX Survey of vectors transmitting Wuchereria bancrofti in Xichang village, Tengxian county, Shandong province. Selection of the National Conference of Parasitic Diseases in 1958 1959 Beijing: The People’s Medical Publishing House 457 460

[B18] Zhang BH Lin JH Zhao YX Studies on the filariasis infection in the residents in Hainan Island and the mosquito vectors. Papers of Filariasis Control and Research in China 1990 Fuzhou: Fujian Publishing House of Science and Technology 25 29

[B19] Miao JW Liu WD Studies on natural infection with Brugia malayi and the bionomics relation to climate of Anopheles hyrcanus sinensis in Linhai, Zhejiang province Acta Entomol Sin 1962 11 4 363 369

[B20] Li XP Chen SY Yu ZQ Epidemiological study on the filariasis in Guangxi I. Survey of the distribution of filariasis and its mosquito vector species. Papers of Filariasis Control and Research in China 1990 Fuzhou: Fujian Publishing House of Science and Technology 13 20

[B21] Gong JZ Xu AN Zhang CL Epidemiological survey of filariasis in Zhenze county, Jiangsu province II. Surveys of vector mosquitoes, especially the relationship between the types of Anopheles sinensis and filariasis transmission. Selection of the national Conference of Parasitic Diseases in 1958 1959 Beijing: The People’s Medical Publishing House 450 457

[B22] Zhang BH Studies on bionomics of different types of Anopheles sinensis and its transmission in brugian filariasis endemic areas Chin Med J 1964 50 12 776 779

[B23] Xu JJ Feng LZ Studies on the Anopheles hyrcanus Group of mosquitoes in China Acta Entomol Sin 1975 18 1 77 98

[B24] Tao ZH Zheng HJ He GB Cheng WF Li MG Wang SH Chen X Fang RL Epidemiologic characteristics and control of filariasis in Guizhou province J Parasitol Parasit Dis 1985 3 4 244 247 3915729

[B25] Xu JJ Zhu HP Luo XF Studies on the susceptibility of A. anthropophagus to experimental infection with Wuchereria bancrofti Chin J Parasitol Parasit Dis 1993 11 2 112 115 8174212

[B26] Zhu HP Fan HJ Observations on natural infection of Wuchereria bancrofti in Anopheles anthropophagus Chin J Parasit Dis Control 1994 7 2 101 103

[B27] Feng LZ Filariasis and control problems Chin Med J 1962 48 2 265 271

[B28] Sun DJ The achievements, strategies and prospects in filariasis control in China Chin J Parasi Dis Control 1990 3 3 187 189

[B29] Chen JT Cui ZH Li WB Comparative studies on integrated measures and case treatment in control of bancroftian filariasis. Papers of Filariasis Control and Research in China 1990 Fuzhou: Fujian Publishing House of Science and Technology 256 260

[B30] Li XP Gan YC Pan SX Observation on effect of different intervention measures against bancroftian filariasis. Papers of Filariasis Control and Research in China 1990 Fuzhou: Fujian Publishing House of Science and Technology 254 256

[B31] Yu JY Twenty seven years’ longitudinal surveillance after basic elimination of filariasis in Hongshang Xiang,Wuhan city. Papers of Filariasis Control and Research in China 1990 Fuzhou: Fujian Publishing House of Science and Technology 321

[B32] Wang Z Control of Lymphatic Filariasis in China 1997 Beijing: The People’s Medical Publishing House 71 112

[B33] Department of Filariasis Shandong institute of parasitic diseases: experimental observation of diethylcarbamazine-medicated salt on the efficacy of filariasis control Chin Med J 1975 55 5 316 318

[B34] National Technical Steering Group for Filariasis Control and Research A great success in lymphatic filariasis control in China Chin J Parasitol Parasit Dis 1995 13 2 81 85 7554168

[B35] Sun DJ Tao ZH Diethylcarbamazine-medicated salt in filariasis control Chin Med J 1979 59 6 374 376

[B36] Liu XJ Liu JY Efficacy of diethylcarbamazine-medicated salt-products for controlling filariasis in Fujian province Acta Fujian Med Coll 1987 21 3 235 237

[B37] Zhong CH Elimination of filariasis in Shangdong province Chinese Journal of Preventive Medicine 1984 18 5 260 263 6537297

[B38] Shi ZJ Sun DJ Achievements in the research on filariasisn in China the past 50 years Chin J Parasitol Parasit Dis 1999 17 5 267 270 12563852

[B39] National Technical Steering Group for Filariasis Control and Research Recent advances in filariasis control in China J Parasitol Parasit Dis 1986 4 4 244 245 3568299

[B40] Collaborating research group on the transmission threshold of filariasis Study on the transmission threshold of filariasis Chin J Parasitol Parasit Dis 1994 12 1 1 6 8044896

[B41] Shi ZJ Study on the transmission threshold of filariasis Chin J Parasitol Parasit Dis 1994 12 1 1 6 8044896

[B42] Duan JH Sun DJ Progress in surveillance and elimination filariasis in China Chin J Parasit Dis Control 2002 15 2 80 81

[B43] Wu WP Shi ZJ Sun DJ A sustainable effort for achieving elimination of lymphatic filariasis in China Chin J Parasitol Parasit Dis 2003 21 6 321 322 15108538

