# Supplementary material for: The history of the elimination of lymphatic filariasis in China
Source: Infect Dis Poverty. 2013 Dec 2;2:30. doi: 10.1186/2049-9957-2-30 (PMC4177192; doi:10.1186/2049-9957-2-30)
Supplement: Additional file 1 — Multilingual abstracts in the six official working languages of the United Nations. [file 2049-9957-2-30-S1.pdf]

Translation of the abstract into the six official working languages of the United Nations

تاريخ القضاء على مرض الخيطيات اللمفاوية في الصين

صن دي جيان، دنغ شو لى، دوان جي هوى

موجز

كانت جمهورية الصين الشعبية واحدة من أكثر البلدان الموبوءة بداء الخيطيات اللمفاوية في العالم. فقد توطن المرض في 864 مقاطعة/مدينة في 16 إقليم/منطقة ذاتية الحكم/بلدية بإجمالي عدد سكان 330 مليون كانوا معرضين للإصابة. وضعت الحكومة الصينية السيطرة على المرض على رأس أولوياتها منذ تأسيس جمهورية الصين الشعبية في عام 1949. وبعد عقود من الجهود المتواصلة، والتعاون الوثيق بين الدوائر الحكومية لمكافحة مرض الخيطيات اللمفاوية، علاوة على المشاركة الفعالة للسكان في المناطق التي ينتشر بها المرض، تم شن حملة قوية وشاملة تكللت بالنجاح للوقاية من هذا المرض ومكافحته. فعلى مدى سنوات عديدة تحققت إنجازات هائلة بسبب الجهود المستمرة للعلماء الصينيين والعاملين في مكافحة المرض، وتم إنجاز الهدف النهائي بالقضاء على مرض الخيطيات اللمفاوية في البلاد في عام 2006.

Translated from English version into Arabic by Ahmed Ahmed, through

TRANSLATORS
WITHOUT BORDERS

中国消除淋巴丝虫病的历史

孙德建，邓绪礼，段绩辉

摘要

中国曾是世界上淋巴丝虫病流行最严重国家之一。该病的流行遍及 16 个省、自治区、直辖市的 864 个县（市、区），有受威胁人口 3.3 亿。1949 年新中国成立以来，中国政府将淋巴丝虫病列为重点防治的危害人民健康最严重的疾病之一。几十年来，在政府的高度重视和关怀下，在有关部门的密切配合和流行区人民群众的积极参与下，全国开展了轰轰烈烈的防治工作。依靠几代科研、防治工作者的不懈努力，到 2006 年实现了全国消除淋巴丝虫病的目标。

Translated from English version into Chinese by Sun De-jian, through

Histoire de l'éradication de la filariose lymphatique en Chine

Sun De-jian, Deng Xu-li, Duan Ji-hui

Résumé

Il fut un temps où la République Populaire de Chine (R.P. Chine) était l'un des pays où la filariose lymphatique (FL) était la plus endémique dans le monde. On y comptait 864 circonscriptions/villes endémiques réparties dans 16 provinces/régions autonomes/municipalités (P/RA/M) pour une population totale de 330 millions d'individus exposés à un risque d'infection. Depuis la création de la R.P. Chine en 1949, le gouvernement chinois a fait de la lutte contre cette maladie une priorité absolue. Des décennies d'efforts soutenus, une coopération étroite des ministères en faveur de la lutte contre la FL et une participation active des populations endémiques ont permis de réaliser une campagne globale de prévention et de lutte menée avec vigueur et efficacité. Au fil des ans, d'importantes avancées ont été réalisées grâce à la persistance des efforts des scientifiques et des agents chinois chargés de la lutte contre la maladie. L'objectif ultime d'éradication de la FL dans le pays a été atteint en 2006.

Translated from English version into French by Eric Ragu, through

История ликвидации лимфатического филяриатоза в Китае

Сан Де-Цзянь, Дэн Сюй-Ли, Дуань Цзи-Хуэй

Резюме

Китайская Народная Республика (КНР) была одной из стран, наиболее подверженных эндемической заболеваемости лимфатическим филяриатозом (ЛФ). Территория риска охватывала 864 города и поселка, 16 провинций, автономных районов и муниципалитетов с общим населением 330 миллионов человек. С самого основания КНР в 1949 году борьба с ЛФ была одним из приоритетов китайского правительства. В результате многолетних усилий, тесного взаимодействия государственных ведомств и при активном участии населения эндемических районов комплексная программа по предотвращению и контролю этого заболевания увенчалась успехом. В течение десятилетий, благодаря настойчивой работе китайских ученых и санитарно-эпидемических служб был пройден огромный путь. Конечная цель – полная ликвидация ЛФ в стране – была достигнута в 2006 году.

Translated from English version into Russian by Leila Usmanova, through

TRANSLATORS
WITHOUT BORDERS

Historia de la eliminación de la filariasis linfática en China

Sun De-jian, Deng Xu-li, Duan Ji-hui

Resumen

La República Popular China (R. P. China) solía ser uno de los países del mundo con mayor nivel de endemia para la filariasis linfática (FL). Había 864 condados/ciudades endémicos en 16 provincias/regiones autónomas/municipios (P/A/M) con una población total de 330 millones en riesgo de infección. Desde la fundación de la R. P. China en 1949, el gobierno chino designó el control de esta enfermedad como una prioridad absoluta. Gracias a décadas de esfuerzo sostenido, de colaboración estrecha en relación con el control de la FL entre los distintos departamentos gubernamentales y de participación activa de las poblaciones endémicas, se ha llevado a cabo con éxito una enérgica campaña global para la prevención y el control de la FL. A lo largo de muchos años se han realizado grandes logros mediante los esfuerzos persistentes de científicos y profesionales del control de las enfermedades chinos. El objetivo final de eliminar la FL en el país se logró en 2006.

Translated from English version into Spanish by Elena de Terán Bleiberg, through

TRANSLATORS
WITHOUT BORDERS
